# Operational intelligence for institutional processes: dynamic modeling and state-based decision policies

**DOI:** 10.3389/frai.2026.1862728

**Published:** 2026-06-29

**Authors:** William Villegas-Ch, Joselin Garcia-Ortiz, Christian Aristizábal, Diego Buenaño-Fernandez

**Affiliations:** 1 Escuela de Ingeniería en Ciberseguridad, Facultad de Ingeniería y Ciencias Aplicadas, Universidad de Las Américas, Quito, Ecuador; 2 Dirección de Investigación y Vinculación, Universidad de Las Américas, Quito, Ecuador

**Keywords:** event-driven dynamic systems, operational adaptability in institutional systems, operational intelligence, process mining, state-based decision making

## Abstract

The management of research processes in higher education institutions is characterized by fragmented operational flows, manual validations, and static decision criteria, resulting in cumulative latency and limited adaptive capacity amid changing workloads. Although artificial intelligence technologies have been incorporated into institutional management, their application remains focused on isolated automation or predictive analysis, without explicitly integrating events, states, and decisions within a unified operational structure. This work proposes an operational intelligence framework in which institutional processes are modeled as a coupled dynamic system formalized as *S*_*t*+1_ = *T*(*S*_*t*_, *E*_*t*_, *D*_*t*_). From operational logs, state trajectories are reconstructed, and two decision policies are evaluated: a deterministic rule-based baseline and a multivariable state-dependent policy. The evaluation is performed using operational metrics oriented toward system dynamics, including decision rate, activation variance, state sensitivity, and transition behavior. The results show that the proposed policy reduces the decision rate from 0.1427 to 0.1002 and the activation variance from 0.3689 to 0.1903, while increasing state sensitivity to 1.0. Under high-load conditions, the model generates transitions with average magnitudes of −777.79, compared to −60.17 in the baseline formulation, while short trajectories exhibit highly selective interventions with stabilization rates of 1.0. These results demonstrate a reconfiguration of the operational decision regime through selective state-dependent interventions.

## 1 Introduction

Research management in higher education institutions operates within environments characterized by heterogeneous operational processes, including project approval, researcher evaluation, financial administration, and institutional request management. Although many of these processes have undergone partial digitalization, their execution still relies on sequential workflows, manual validation, and fragmented decision criteria, resulting in cumulative latency, operational redundancy, and variability in response times. Previous studies have shown that workflow-based systems in university environments continue to face structural limitations associated with process integration and organizational coordination (Benavides et al., 2020). At the same time, automation-oriented approaches have mainly focused on reducing administrative workload and processing times (Boza-Chua et al., 2022). Recent literature further identifies administrative burden, process fragmentation, and limited interoperability as persistent constraints affecting operational efficiency in higher education institutions (Guarda et al., 2025). In this scenario, the problem extends beyond the availability of digital technologies. It reflects the absence of a formal operational model that explicitly integrates events, states, and decisions into a single dynamic structure.

Existing approaches remain fundamentally limited in their capacity to represent institutional operations as integrated dynamic systems. Workflow management systems prioritize process structure and traceability without modeling adaptive decision-making behavior; robotic process automation relies on predefined rules without contextual operational reasoning; and artificial intelligence is frequently incorporated as an analytical or predictive layer, separated from the operational flow. Consequently, current frameworks do not provide a unified representation that jointly models interactions among events, reconstructed operational states, and decision-generation processes over time.

From a systems perspective, institutional environments often operate through partially decoupled information flows, in which processes evolve without coordinated, event-driven orchestration. This behavior has been documented in university management systems where, despite the adoption of workflow platforms and integrated administrative tools, technical and organizational constraints continue to limit sustained improvements in operational coordination (Benavides et al., 2020). Under these conditions, critical operational decisions, including budget validation, request prioritization, and workload distribution, are often determined by static rules or implicit human criteria, limiting the system's capacity to adapt to changes in operational demand. In addition, the coexistence of multiple non-integrated digital systems introduces redundancies in information management and limits the construction of coherent operational ecosystems (Kovalenko et al., 2021). Although recent advances in automation and artificial intelligence aim to improve organizational efficiency, their adoption in educational institutions continues to face challenges stemming from operational heterogeneity, organizational complexity, and fragmented decision-making processes.

Given these limitations, there is a need for a formal framework that can represent institutional operations as coupled dynamic systems in which events, operational states, and decisions evolve jointly. This study proposes an operational intelligence framework in which organizational processes are modeled through dynamic state transitions governed by the interaction between observable events and decision policies. The proposed formulation enables institutional operations to be represented as evaluable temporal trajectories, in which each decision directly contributes to the evolution of the operational state. Unlike existing approaches that treat process representation, state modeling, and decision generation as separate analytical components, the proposed framework formalizes their interaction within a unified operational structure. Operational trajectories are reconstructed directly from observable event histories, enabling decisions, state transitions, and operational evolution to be analyzed as coupled elements of a single dynamic process.

Methodologically, the proposed approach is based on operational reconstruction from event logs, transforming temporal interaction sequences into structured trajectories that preserve the temporal dependencies of the original processes. This reconstruction mechanism enables the derivation of operational states from observable event histories while preserving traceability between raw interactions and generated decisions. The framework is evaluated using two complementary datasets: EdNet-KT1, composed of high-frequency educational interaction logs, and BPI Challenge 2017, composed of organizational process traces with case-level temporal evolution. Based on the reconstructed trajectories, two decision policies are evaluated: a deterministic rule-based baseline and a multivariable state-dependent policy. The evaluation focuses on operational metrics associated with system dynamics, including decision rate, activation variance, state sensitivity, transition behavior, and stabilization dynamics.

The results show that the proposed policy does not uniformly reduce operational complexity; instead, it reorganizes the system's decision regime according to the structure of the reconstructed state space. Quantitatively, the proposed formulation reduces the decision rate from 0.1427 to 0.1002 and decreases activation variance from 0.3689 to 0.1903, indicating lower global dispersion in activation behavior. At the same time, state sensitivity increases to 1.0, demonstrating that decisions become strongly dependent on specific operational configurations. Under high-load conditions, the proposed policy generates transitions that reach magnitudes of −777.79, compared to −60.17 in the baseline model, while short trajectories exhibit highly selective interventions with strong stabilization effects.

The main contribution of this work lies in formalizing operational intelligence as a measurable, reproducible dynamic system in which events, states, and decisions are jointly evaluated using observable operational metrics. By integrating state reconstruction, sequential event dynamics, and state-dependent decision policies into a single framework, the proposed model overcomes the traditional separation among workflow automation, predictive analytics, and reactive processing architectures.

## 2 Literature review

The evolution of operating systems in organizational environments, particularly in higher education institutions, has been influenced by various technological paradigms, including business process management (BPM), robotic process automation (RPA), AI, event-driven architecture, and multi-agent systems. While these approaches have demonstrated improvements in efficiency, scalability, and decision support, the literature reveals a persistent fragmentation that limits their integration into adaptive systems.

From a structural perspective, BPM-based approaches and business intelligence (BI) ecosystems have enabled the formalization of processes and the integration of organizational data. Recent studies report improvements in traceability, interdepartmental coordination, and administrative efficiency through structured workflows and integrated information systems (Suazo-Galdamés and Chaple-Gil, 2025; Zhang et al., 2024), as well as advances in data-driven decision-making in higher education (Wu et al., 2024; Sequeira et al., 2025). However, these systems primarily operate under retrospective analytical frameworks, which limit their ability to adapt to changing operational dynamics and evolving interaction patterns in real time.

In this context, RPA has emerged as a solution for automating repetitive administrative tasks, thereby reducing operational workload and processing times across various institutional processes (Guarda et al., 2025). It also contributes to improving consistency and accuracy in the execution of standardized tasks (Al-Maadeed et al., 2021; Suazo-Galdamés and Chaple-Gil, 2025). However, its reliance on rigid rules limits its performance in dynamic, unpredictable environments where operational conditions evolve continuously.

Complementarily, AI and machine learning have been incorporated into organizational decision-making, demonstrating improvements in predictive accuracy, resource optimization, and strategic support (Pantin, 2026; Zhang et al., 2024). These approaches enable the analysis of large volumes of data and the generation of actionable knowledge, thereby strengthening institutional intelligence (Kadhem, 2025; Silveira et al., 2026), with reported impacts on operational efficiency in educational settings (Suazo-Galdamés and Chaple-Gil, 2025). Recent studies have also explored adaptive monitoring and intelligent decision-support mechanisms in dynamic systems using integrated learning architectures and data-driven operational modeling (Ngnassi Djami and Abdelhakim, 2026; Ngnassi Djami and Nzié, 2025). Despite these advances, most AI-driven approaches remain centered on predictive optimization or domain-specific operational monitoring, without explicitly modeling the coupled interaction between events, reconstructed states, and operational decisions within a unified temporal framework.

Event-driven architectures have been proposed to manage reactive systems by processing continuous data streams in real time, thereby improving system responsiveness (Eshankulov et al., 2026; Sowan et al., 2025). Furthermore, integration with BI and advanced analytics systems enhances continuous processing and information availability (Wu et al., 2024; Sequeira et al., 2025). However, these approaches do not incorporate explicit mechanisms for adaptive decision-making based on reconstructed operational states.

At a higher level, multi-agent systems enable distributed decision-making through the interaction of multiple entities, demonstrating adaptive and resilient capabilities in dynamic environments (Basingab, 2025). These systems facilitate coordinated local decision-making in highly distributed scenarios (Peng et al., 2024), although their integration with structured organizational processes remains limited (Kadhem, 2025). Existing formulations also tend to separate reactive behavior, operational state representation, and decision execution into independent analytical layers, limiting the direct evaluation of system evolution under coupled operational dynamics.

The literature shows that current approaches address key dimensions such as structure, automation, intelligence, reactivity, and autonomy in isolation, without achieving coherent integration into a unified model. This fragmentation reveals a critical gap: the absence of a formal framework for modeling operating systems as dynamic entities in which states, events, and decisions are intrinsically linked. This limits the analysis of their evolution and operational properties such as latency, variability, adaptation, and decision consistency. Overcoming this limitation requires moving toward an integrated operational intelligence framework that formalizes the relationships among event flows, reconstructed states, and adaptive decision policies within a unified, evaluable dynamic structure, which constitutes the basis of the model proposed in this work.

## 3 Materials and methods

### 3.1 Formulation of the operational intelligence system

The operational intelligence system is formulated as a discrete dynamic process in which the system behavior evolves through sequential interactions observed over time. The formulation integrates events, reconstructed states, and decision functions within a unified temporal structure, enabling the representation of operational evolution as a coupled process rather than as isolated observations or static process snapshots.

Let *E* = {(*e*_*i*_, *t*_*i*_)} be the set of timestamped events, where each event *e*_*i*_ corresponds to an elementary interaction generated by the system at time *t*_*i*_. Each event contains observable operational attributes, including interaction type, execution duration, temporal occurrence, and contextual process information. The subset *E*_≤ *t*_ represents the ordered event history available up to time *t*, preserving the sequential dependencies required for dynamic reconstruction.

The operational state is not directly observable in the raw event log and is therefore reconstructed through a deterministic aggregation process applied to the accumulated event history. The state representation is defined in Equation 1:


$$\begin{array}{l} S_{t}=\phi \left(E_{\leq t}\right) \end{array}$$ (1)

where $\phi :{\mathbb{R}}^{\left|E_{\leq t}\right|}\to {\mathbb{R}}^{d}$ defines a mapping from the ordered event space to a *d*-dimensional operational state space. The transformation ϕ(·) is instantiated by applying cumulative and temporal aggregation operators to sequential observations, generating derived variables associated with operational intensity, interaction recurrence, elapsed-time accumulation, and local behavioral statistics computed over sliding temporal windows. This reconstruction mechanism allows the state representation to evolve incrementally as new events are incorporated into the trajectory.

Decision generation is formalized as a policy dependent on the reconstructed operational state and the incoming event, as defined in Equation 2:


$$\begin{array}{l} D_{t}=\pi \left(S_{t},E_{t}\right) \end{array}$$ (2)

where π:ℝ^*d*^×*E*→*D* represents the operational decision policy. The policy evaluates the current state configuration and the characteristics of the incoming event to determine whether an intervention or a decision activation should occur. In the proposed formulation, the policy operates exclusively on variables reconstructed from the event stream without incorporating exogenous information or external supervisory signals. This restriction preserves traceability between observed events, reconstructed states, and generated decisions throughout the operational sequence.

The system dynamics are modeled through a transition function that updates the reconstructed state after each interaction, as defined in Equation 3:


$$\begin{array}{l} S_{t+1}=T\left(S_{t},E_{t},D_{t}\right) \end{array}$$ (3)

where *T* represents the transition operator governing the evolution of the operational state, the transition process incorporates the current state, the incoming event, and the generated decision, allowing the system trajectory to evolve under temporally dependent interactions. This formulation preserves sequential continuity between states and enables the representation of feedback effects from operational decisions over time.

The reconstructed states are derived deterministically from observable event histories, allowing the transition process to represent operational evolution through successive event-driven updates of the state space. Under this formulation, state transitions emerge from the interaction between observed events, reconstructed operational conditions, and generated decisions, preserving complete traceability throughout the trajectory. This representation enables direct analysis of how decision behavior influences operational evolution over time while maintaining explicit links between event-level activity, reconstructed states, and resulting operational outcomes.

To preserve temporal consistency and entity-level traceability, the operational process is represented through ordered trajectories associated with each entity *k*, as defined in Equation 4:


$$\begin{array}{l} \tau^{\left(k\right)}=\left\{\left(E_{1},S_{1}\right),\left(E_{2},S_{2}\right),\ldots ,\left(E_{T},S_{T}\right)\right\} \end{array}$$ (4)

where each trajectory τ^(*k*)^ corresponds to an ordered sequence of events and reconstructed states associated with a single operational entity. This structure enables longitudinal analysis of state evolution, operational variability, and decision behavior across heterogeneous interaction regimes and temporal conditions.

### 3.2 Data sources and event structure

Two complementary data sources are used in this study: the EdNet-KT1 dataset, which contains high-frequency student-system interaction sequences, and the BPI Challenge 2017 dataset, which contains event logs derived from real organizational processes with case-level traceability and implicit operational decisions. Both datasets are incorporated as sources of observable events, preserving their original temporal structure and avoiding synthetic generation or external reconstruction procedures.

In the EdNet-KT1 dataset, each event corresponds to an elementary interaction generated during the user learning process, as defined in Equation 5:


$$\begin{array}{c} E_{t}^{(u)}=({\text{timestamp}}_{t},{\text{solving\_id}}_{t},\\ {\text{question\_id}}_{t},{\text{user\_answer}}_{t},{\text{elapsed\_time}}_{t}) \end{array}$$ (5)

where *u* denotes the user identifier and *t* represents the temporal index associated with the interaction sequence. Each event, therefore, contains both temporal and operational attributes that describe the progression of the interaction process.

For the BPI Challenge 2017 dataset, events are represented as organizational process transitions associated with individual cases, as defined in Equation 6:


$$\begin{array}{l} E_{t}^{\left(c\right)}=({\text{case\_id}}_{t},{\text{concept:name}}_{t},\\ {\text{lifecycle:transition}}_{t},{\text{timestamp}}_{t}) \end{array}$$ (6)

where *c* identifies a process case, and each event represents a discrete transition within the operational workflow. Although the semantic interpretation of the events differs across domains, both datasets preserve the same structural representation: temporally ordered observations associated with identifiable entities.

Conceptual normalization across datasets is achieved by defining a common analytical unit based on entity-centered event trajectories. In EdNet-KT1, the entity corresponds to the user, and the event represents an interaction with the educational platform. In BPI 2017, the entity corresponds to the organizational case, and the event represents a process transition within the workflow. In both scenarios, each event modifies the temporal trajectory of the associated entity and contributes incrementally to the reconstruction of its operational state.

To formalize this correspondence, a temporal ordering operator *O* is defined to induce a sequential structure over the events associated with each entity, as defined in Equation 7:


$$\begin{array}{c} E^{\left(k\right)}=O\left(\left\{E_{i}^{\left(k\right)}\right\}\right)\\ =\left\{E_{1}^{\left(k\right)},E_{2}^{\left(k\right)},\ldots ,E_{T}^{\left(k\right)}\right\},\;t_{1}<t_{2}<\cdots <t_{T} \end{array}$$ (7)

where *k* represents the operational entity, corresponding either to a user or to a process case, depending on the dataset, the ordering process preserves temporal consistency and guarantees that the event sequence maintains the original chronological dependencies observed in the source system.

Under this formulation, both datasets are interpreted as realizations of a common event-driven dynamic process in which semantic differences remain encapsulated within the event attributes specific to each domain. This abstraction preserves structural consistency across heterogeneous sources while allowing the operational intelligence framework to model educational and organizational processes under a unified sequential representation.

### 3.3 Operational reconstruction of the dataset

**Figure 1:**
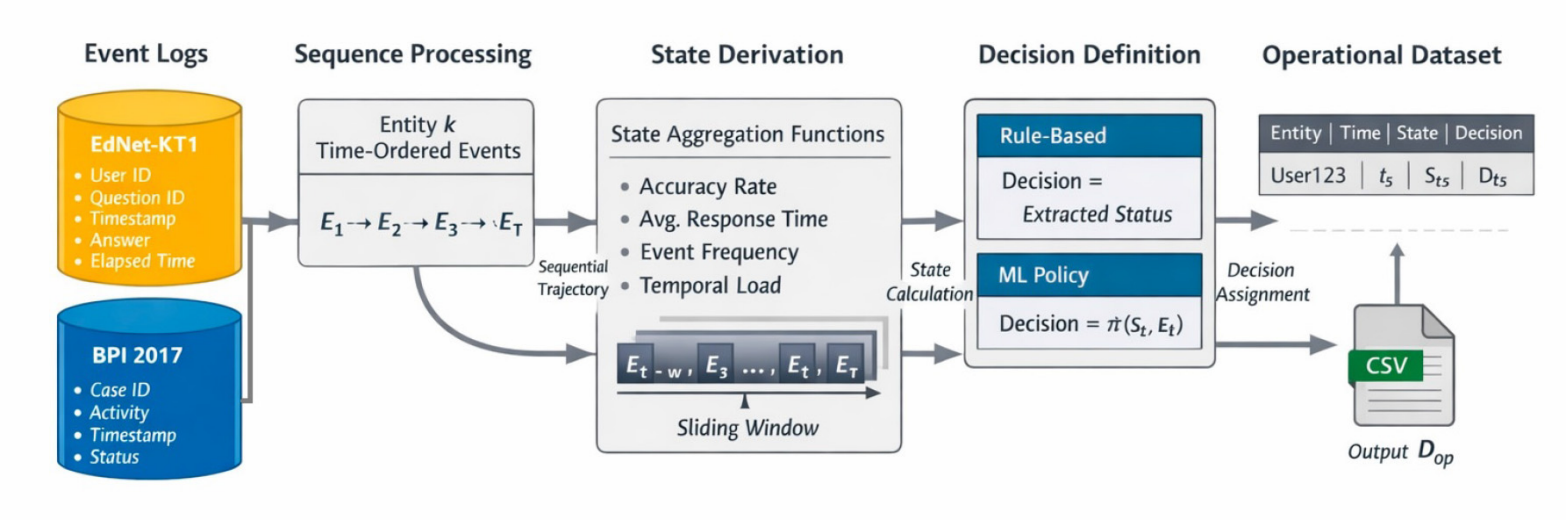
Operational dataset reconstruction pipeline from event logs.

The operational dataset is reconstructed from the original event logs through a sequential transformation process that integrates temporal ordering, state derivation, and decision assignment. This reconstruction converts heterogeneous event streams into structured operational trajectories that preserve the temporal dependencies required for dynamic analysis. Figure 1 illustrates the complete reconstruction pipeline from the raw event logs to the generation of the operational dataset.

Initially, events are grouped according to their associated entity *k* and ordered chronologically using their timestamp information, generating sequential trajectories as defined in Equation 8:


$$\begin{array}{l} E^{\left(k\right)}=\left\{E_{1}^{\left(k\right)},E_{2}^{\left(k\right)},\ldots ,E_{T}^{\left(k\right)}\right\} \end{array}$$ (8)

where *k* corresponds to the user in EdNet-KT1 and to the process case in BPI 2017, this ordering process preserves the temporal continuity of interactions. It enables reconstruction of the operational evolution associated with each entity. In EdNet-KT1, trajectories represent sequences of educational interactions generated during user activity, whereas in BPI 2017, they represent progressive transitions within organizational workflows.

After temporal sequencing, state variables are derived from the ordered trajectories using temporal aggregation operators applied over cumulative histories and sliding windows of size *w*. The reconstructed state at time *t* is defined in Equation 9:


$$\begin{array}{l} S_{t}=\phi \left(E_{t-w:t}\right) \end{array}$$ (9)

where ϕ(·) transforms the local event history into a structured operational representation. In the experimental implementation, the transformation generates variables for accumulated interaction time, inter-event intervals, operational intensity, interaction recurrence, and local temporal statistics computed over sliding windows of sizes 3, 5, and 10 events. This mechanism allows the reconstructed state to evolve incrementally as new observations are incorporated into the trajectory.

The operational dataset is generated iteratively for each trajectory. At each temporal step *t*, the current event *E*_*t*_ is processed together with the reconstructed state *S*_*t*_, and a decision variable *D*_*t*_ is assigned according to the operational characteristics of the dataset. In the BPI 2017 dataset, decisions are extracted directly from process attributes associated with the workflow transitions. In EdNet-KT1, decisions are generated through the policy π(*S*_*t*_, *E*_*t*_) using only variables reconstructed from the event sequence.

The reconstruction procedure is formalized through the computational scheme presented in Algorithm 1.

Algorithm 1 Construction of the operational dataset. 
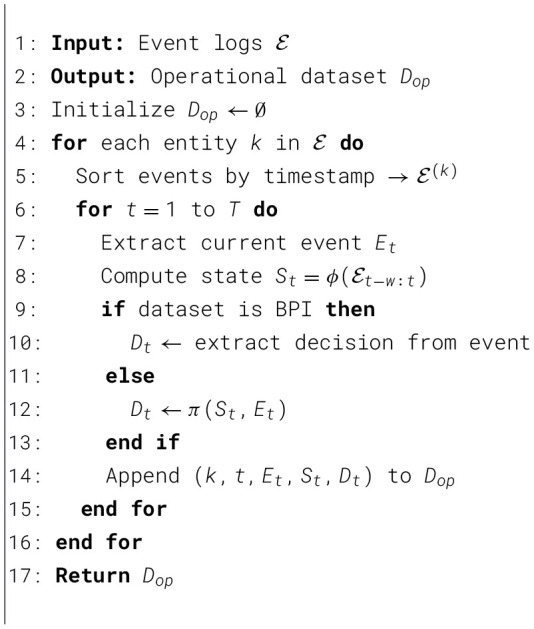

The application of this procedure to EdNet-KT1, using a sample of 5,000 users, yielded 1,402,425 valid events after preprocessing and temporal filtering, along with the identification of 79,242 sessions, differentiated by temporal discontinuities between consecutive interactions. The reconstruction process generated a state space comprising 28 operational variables per event, capturing temporal accumulation, operational intensity, interaction recurrence, and local behavioral dynamics across trajectories. The resulting interaction structure exhibited an average of 280 events per user and a median of 30 events per user, reflecting substantial heterogeneity in the operational trajectories reconstructed from the event logs.

### 3.4 Decision policy modeling

**Figure 2:**
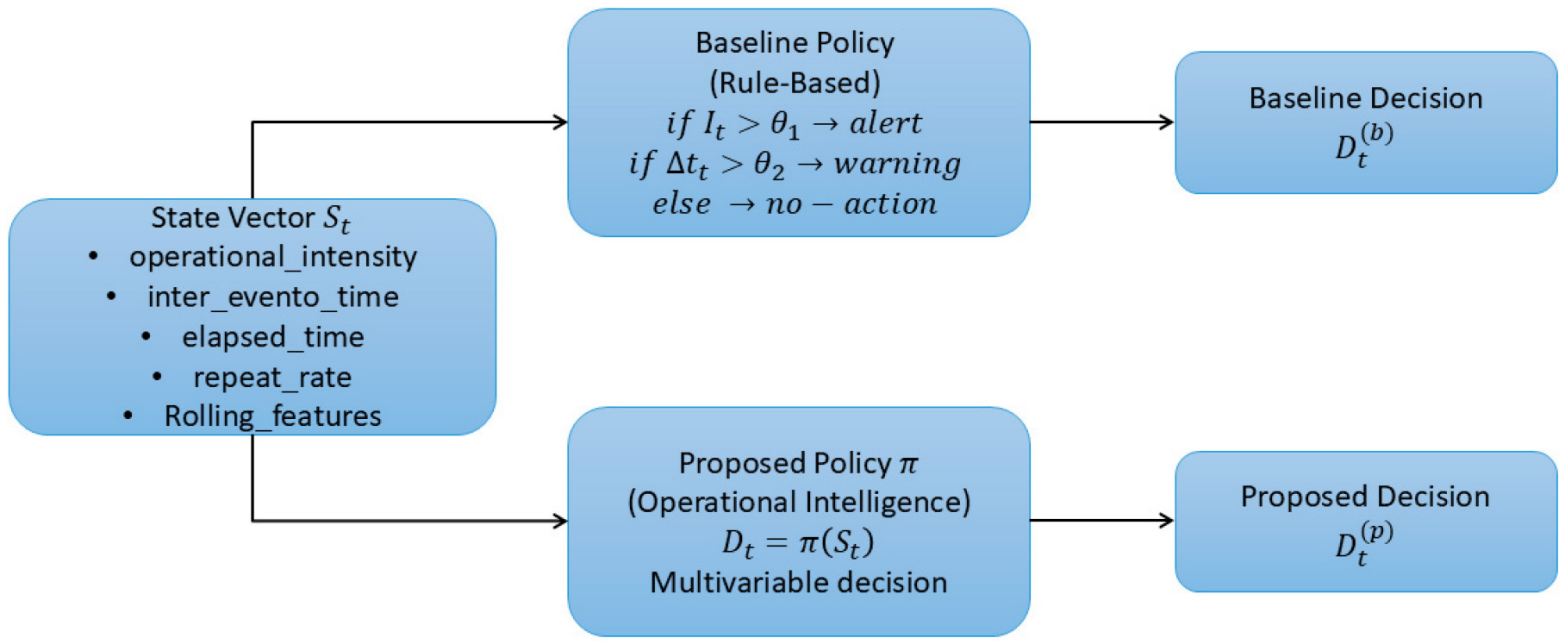
Decision policy modeling framework.

Decision-making is formulated as a state-dependent process in which each action is generated exclusively from the reconstructed operational representation derived from the event trajectories. Figure 2 presents the general decision architecture, where the state vector *S*_*t*_ serves as the common input for both the rule-based baseline policy and the proposed multivariable operational intelligence model.

The reconstructed state *S*_*t*_ contains multivariate information describing the operational condition of the system at time *t*. This representation integrates variables associated with operational intensity, accumulated temporal load, inter-event intervals, interaction recurrence, and local temporal statistics derived from sliding windows. The decision mechanism is defined directly over the reconstructed state space, as shown in Equation 10:


$$\begin{array}{l} D_{t}=\pi \left(S_{t}\right) \end{array}$$ (10)

where π represents a decision policy operating over the derived operational state, under this formulation, all generated decisions remain fully traceable to observable event sequences and reconstructed state variables, avoiding the incorporation of exogenous information or external supervisory inputs.

This formulation also provides an interpretable operational decision mechanism in which each generated action can be traced directly to observable state variables and temporally reconstructed event trajectories. Unlike operational models based on latent or non-transparent representations and opaque optimization processes, the proposed framework preserves structural interpretability by maintaining explicit relationships among operational variables, reconstructed states, activation conditions, and resulting decisions. Consequently, the contribution of operational dimensions such as temporal accumulation, interaction recurrence, operational intensity, and local behavioral variability can be analyzed directly within the decision-generation process. This property enables transparent state-transition reasoning, retrospective auditing of operational activations, and explainable operational policy behavior under evolving institutional conditions while preserving consistency with the observed event dynamics. This property is particularly relevant in institutional operational environments where transparency, auditability, and decision accountability are required for operational supervision and governance.

A deterministic rule-based policy is initially implemented as a baseline reference model. This policy generates decisions by applying threshold conditions independently to individual state variables, thereby establishing an interpretable activation mechanism directly associated with observable operational metrics. The baseline formulation uses variables such as operational intensity *I*_*t*_ and inter-event time Δ*t*_*t*_ to identify predefined activation conditions associated with high operational load or irregular temporal behavior.

The thresholds and decision boundaries used in the baseline policy are derived empirically from the distributions of the reconstructed variables. This procedure preserves consistency with the observed operational behavior while maintaining the policy entirely data-driven. However, because the baseline evaluates variables independently, the resulting decisions cannot explicitly model interactions among multiple operational dimensions that evolve simultaneously across trajectories.

To address this limitation, a multivariable operational intelligence policy is defined in which the decision process integrates the complete reconstructed state in a unified manner. Instead of activating isolated threshold conditions, the proposed formulation evaluates the operational configuration using a global scoring function that captures dependencies among state variables. The policy is defined in Equation 11:


$$\begin{array}{l} D_{t}=𝕀\left(f\left(S_{t}\right)>\tau \right) \end{array}$$ (11)

where *f*(*S*_*t*_) represents a scoring function operating over the reconstructed state vector, τ corresponds to the activation threshold, and 𝕀(·) denotes the indicator function that transforms the resulting score into a discrete operational decision.

The scoring function is implemented as a weighted aggregation of normalized operational dimensions derived from the reconstructed state representation, as defined in Equation 12:


$$\begin{array}{l} f\left(S_{t}\right)=0.32I_{t}+0.22U_{t}+0.18R_{t}+0.18M_{t}+0.10C_{t} \end{array}$$ (12)

where *I*_*t*_ denotes normalized operational intensity, *U*_*t*_ represents normalized temporal urgency derived from inter-event intervals, *R*_*t*_ corresponds to normalized interaction recurrence rate, *M*_*t*_ denotes a normalized smoothed behavioral signal computed from local temporal windows, and *C*_*t*_ represents normalized cumulative temporal accumulation. All dimensions are normalized to the interval [0, 1] using min-max scaling to ensure comparable contributions across heterogeneous operational variables.

The activation threshold is determined empirically from the distribution of the reconstructed state scores according to Equation 13:


$$\begin{array}{l} \tau =Q_{0.90}\left(f\left(S_{t}\right)\right) \end{array}$$ (13)

where *Q*_0.90_ denotes the 90th percentile of the score distribution. Consequently, operational activations are generated only for states whose scores belong to the highest decile of the reconstructed state distribution.

The scoring function *f*(*S*_*t*_) is constructed exclusively from variables derived from the operational trajectories, including operational intensity measures, temporal accumulation, interaction recurrence, and local behavioral statistics. Consequently, the proposed formulation preserves complete traceability among original event sequences, reconstructed states, and generated decisions, while allowing the policy to adapt to heterogeneous operational conditions by jointly evaluating multiple state dimensions.

Because the scoring mechanism operates exclusively on explicitly reconstructed operational variables, the resulting decision process maintains operational transparency despite the policy's multivariable nature. Each operational activation can be associated with observable variations in the reconstructed state dimensions rather than hidden latent representations or non-transparent optimization layers. This characteristic enables explainable operational policies, in which decision behavior can be analyzed by examining the contributions of operational intensity, temporal accumulation, interaction recurrence, and local trajectory dynamics within the global scoring configuration. As a result, the framework supports transparent operational auditing and interpretable adaptive behavior while preserving direct traceability between event-level activity, reconstructed state transitions, and generated operational decisions.

The structural difference between the two approaches lies in how operational information is processed during decision generation. The baseline policy evaluates isolated activation conditions independently for each variable. In contrast, the proposed policy treats the reconstructed state as an integrated operational representation, enabling adaptive responses to complex combinations of temporal and behavioral patterns within the evolving trajectories.

### 3.5 Operational evaluation framework

The operational evaluation is formulated as a comparative analysis of two decision policies applied to the same reconstructed trajectories: a deterministic, rule-based baseline and the proposed multivariable operational intelligence model. Both policies operate on identical event sequences and reconstructed state representations, allowing the observed differences in system behavior to be attributed exclusively to the decision-generation mechanism.

Rather than evaluating predictive accuracy or classification performance, the proposed framework analyzes how decisions affect the system's operational evolution by altering the reconstructed state space. The evaluation, therefore, focuses on the dynamic behavior induced by each policy across the event trajectories.

Three complementary evaluation dimensions are considered: responsiveness, operational stability, and adaptive behavior under varying operational conditions. The evaluation metrics employed combine established analytical concepts with operational measures tailored to state-based institutional process analysis. Decision entropy is derived from information-theoretic principles and quantifies the uncertainty associated with policy activation behavior. Activation variance is based on classical statistical dispersion and measures the variability of decision rates across operational entities. State sensitivity adapts sensitivity-analysis concepts to quantify how decision behavior varies across different regions of the reconstructed state space. Similar-state instability is introduced as an operational consistency measure that evaluates whether states with comparable operational configurations produce homogeneous decision responses. While decision entropy, activation variance, and sensitivity-based measures originate from established analytical traditions, similar-state instability is proposed as an operational metric designed specifically to assess decision consistency within reconstructed state regions. These metrics characterize complementary aspects of operational behavior, including responsiveness, consistency, adaptability, and decision concentration, providing a multidimensional evaluation of policy effects on system evolution.

Responsiveness is evaluated through the frequency, temporal distribution, and activation behavior of the generated decisions. Because decisions are directly associated with the evolution of event trajectories, the analysis measures how each policy responds to changes in operational intensity and temporal accumulation across the reconstructed state space. This allows identification of whether the policy concentrates activation behavior within specific operational regions or distributes decisions uniformly across trajectories.

Operational stability is analyzed through the consistency of decision behavior across similar regions of the reconstructed state space. States with comparable operational configurations are grouped and evaluated to determine whether the policy yields homogeneous responses or exhibits substantial variation under equivalent conditions. This analysis allows assessment of the internal variability of the decision structure and the degree of sensitivity to fluctuations in the local state.

Adaptive behavior is evaluated by the effects of decisions on the evolution of operational state variables. The analysis measures how the generated decisions modify the direction and magnitude of transitions between consecutive states, particularly under conditions associated with elevated operational load, interaction irregularities, or unstable temporal behavior. The state transition variation is formalized as shown in Equation 14:


$$\begin{array}{l} \Delta S_{t}=S_{t+1}-S_{t} \end{array}$$ (14)

where Δ*S*_*t*_ represents the variation between consecutive reconstructed states after the application of decision *D*_*t*_, this representation allows evaluation of whether the policy contributes to stabilization, persistence, or amplification of operational conditions throughout the trajectories. The evaluation is performed over both individual and aggregated trajectories, enabling comparison of policy behavior under equivalent operational conditions while preserving the temporal dependencies present in the original event sequences.

## 4 Results

### 4.1 Dataset characterization and interaction dynamics

The characterization of the EdNet-KT1 set is based on descriptive statistical metrics of the distributions of events and sessions, as well as the temporal dynamics, presented in Table 1.

**Table 1:** Statistical characterization of event distribution and temporal dynamics in EdNet-KT1.

Variable	Mean	Median	SD	P90	P95	P99
Events per user	280.49	30.00	1,047.2	591.10	1,396.4	4,417.1
Sessions per user	15.85	1.00	54.68	36.00	77.00	251.03
Events per session	17.70	10.00	21.65	38.00	52.00	105.00
Session duration (ms)	466,617.9	281,000.0	589,120.5	1,065,000	1,521,945	2,848,578
Inter-event time (ms)	18,164,318	34,043.0	478,725,170	257,368.0	2,574,464	147,940,518

The event activity observed at the user level shows a marked difference between the mean and the median, with values of 280.49 and 30.00, respectively, and a standard deviation of 1,047.2. The upper percentiles reach 591.10 events at P90, 1,396.4 at P95, and 4,417.1 at P99, indicating the presence of trajectories with substantially higher interaction volumes than those observed in most entities. This asymmetry reflects heterogeneous operational histories with different levels of event accumulation across the reconstructed trajectories.

Session-level participation across users exhibits an even more concentrated structure, with a mean of 15.85 and a median of 1.00. The percentiles reach 36.00 at P90, 77.00 at P95, and 251.03 at P99, indicating that a limited subset of entities accumulates recurrent sessions over extended periods. The observed variability, with a standard deviation of 54.68, indicates that session continuity is not uniformly distributed across the operational environment.

Session interaction density presents a mean of 17.70 and a median of 10.00, with percentiles of 38.00, 52.00, and 105.00 for P90, P95, and P99, respectively. Most sessions remain concentrated in low-density interaction ranges, while a smaller subset reaches substantially higher event-accumulation levels. Compared with the variability observed at the user level, the dispersion within sessions remains more constrained, suggesting that the principal source of heterogeneity emerges from the longitudinal accumulation of interactions.

Session duration had a mean of 466,617.9 ms and a median of 281,000.0 ms, with a standard deviation of 589,120.5 ms. The upper percentiles reached 1,065,000 ms at P90, 1,521,945 ms at P95, and 2,848,578 ms at P99, indicating a progressive expansion in the duration of a limited subset of sessions. This temporal variation produces trajectories with different levels of continuity and operational persistence.

Inter-event time showed the greatest divergence among the central measures, with a mean of 18,164,318 ms, a median of 34,043.0 ms, and a standard deviation of 478,725,170 ms. The percentiles reached 257,368.0 ms at P90, 2,574,464 ms at P95, and 147,940,518 ms at P99, reflecting the coexistence of compressed interaction intervals and prolonged inactive periods within the same operational environment. This temporal variability produces irregular interaction frequencies across the reconstructed trajectories.

**Figure 3:**
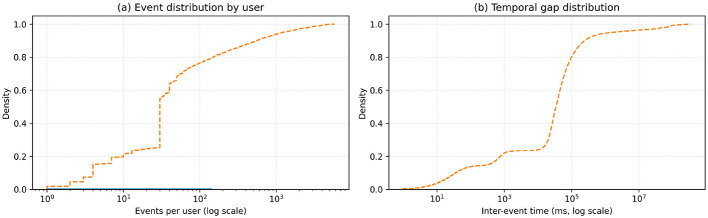
Cumulative distribution of events and temporal dynamics in EdNet-KT1: **(a)** cumulative distribution of events per user on a logarithmic scale; **(b)** cumulative distribution of times between events on a logarithmic scale.

Figure 3 presents the cumulative distribution of events per user and the inter-event times on a logarithmic scale. In Figure 3a, the cumulative event accumulation pattern across users exhibits a non-linear progression throughout the domain. The lower interaction ranges contain a substantial concentration of entities, while the upper regions correspond to a reduced subset of trajectories with significantly higher event volumes. The variation in slope across the logarithmic scale reflects differences in interaction density and trajectory persistence within the operational space.

In Figure 3b, the cumulative temporal accumulation of inter-event intervals shows variations in growth behavior across the temporal domain. Lower ranges are associated with shorter interaction intervals and higher event concentration, whereas larger temporal ranges exhibit slower accumulation due to prolonged pauses between interactions. The resulting structure demonstrates heterogeneous temporal frequencies across the analyzed trajectories.

### 4.2 Operational state space construction and dynamics

The construction of the state $S_{t}=\phi \left(E_{\leq t}\right)$ is analyzed using derived variables that incorporate temporal accumulation, operational intensity, and interaction recurrence. The quantitative characterization of these variables is presented in Table 2.

**Table 2:** Statistical characterization of reconstructed operational state variables in EdNet-KT1.

Variable	Mean	Median	SD	P90	P95	P99
Operational intensity (raw)	3.5696	0.000235	59.6041	0.00245	0.03664	0.06761
Cumulative elapsed time (hours)	2,968.93	1,329.04	3,699.22	8,399.70	10,650.43	15,789.54
Repeat question rate (rolling w = 5)	0.1756	0.0000	0.3439	1.0000	1.0000	1.0000
Smoothed intensity (rolling w = 5)	26,321.33	21,800.0	20,705.71	43,350.0	56,400.0	91,600.0

Operational intensity (raw) shows a marked difference between the mean (3.5696) and the median (0.000235), with a standard deviation of 59.6041. The upper percentiles reach 0.00245 at P90, 0.03664 at P95, and 0.06761 at P99, indicating that most reconstructed states remain concentrated in low-intensity regions, while a smaller subset accumulates substantially higher operational activity levels. The relationship between the mean and the median reflects a highly asymmetric distribution within the reconstructed state space.

Cumulative elapsed time (hours) has a mean of 2,968.93 h, a median of 1,329.04 h, and a standard deviation of 3,699.22 h. The percentiles reach 8,399.70 h at P90, 10,650.43 h at P95, and 15,789.54 h at P99. These values indicate a progressive expansion in the temporal scale of the reconstructed trajectories, in which a reduced subset of entities accumulates substantially longer operational histories than those in the dominant interaction regime.

The repeat question rate variable (rolling *w* = 5) has a mean of 0.1756, a median close to 0, and a standard deviation of 0.3439. The upper percentiles reach 1.0 for P90, P95, and P99, indicating states in which the repetition rate remains at its maximum within the analyzed window. This configuration reflects the coexistence of low-recurrence states and localized regions, with sustained repetition of interactions throughout the reconstructed trajectories.

The smoothed intensity variable (rolling *w* = 5) has a mean of 26,321.33, a median of 21,800.0, and a standard deviation of 20,705.71. The percentiles reach 43,350.0 at P90, 56,400.0 at P95, and 91,600.0 at P99. Compared with the unsmoothed operational intensity, this variable exhibits a more stable distribution across the reconstructed domain, reflecting the effect of temporal aggregation on the scale and continuity of operational activity.

**Figure 4:**
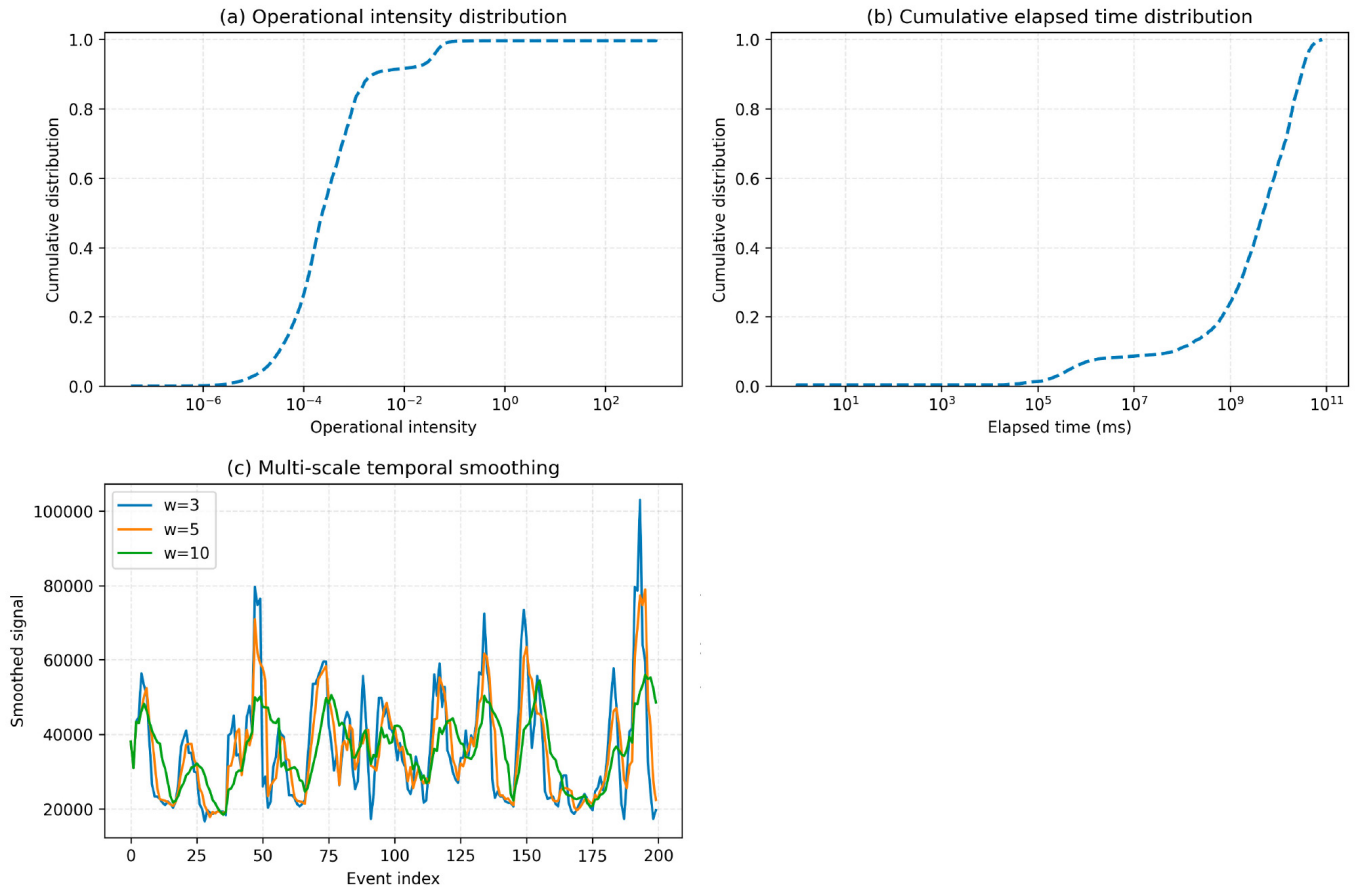
Distribution and structure of the operational state space in EdNet-KT1: **(a)** cumulative distribution of operational intensity on a logarithmic scale; **(b)** cumulative distribution of elapsed time in h on a logarithmic scale; **(c)** non-linear relationship between cumulative time and operational intensity in the state space.

Figure 4 presents the cumulative distribution of state variables and the relationship between components in the reconstructed space. In Figure 4a, the cumulative distribution of operational intensity (raw) remains concentrated within the lower ranges of the logarithmic domain. The accumulation accelerates toward values near zero, indicating that a substantial proportion of reconstructed states exhibit reduced operational intensity. In contrast, higher-intensity states appear with progressively lower frequency across the domain.

In Figure 4b, the cumulative distribution of cumulative elapsed time extends across multiple orders of magnitude on the logarithmic scale. The accumulation progresses more gradually than the operational intensity distribution, reflecting the presence of trajectories with substantially different temporal lengths and persistence levels within the reconstructed state space.

In Figure 4c, the relationship between cumulative elapsed time and operational intensity is represented in the two-dimensional state space. The observations remain concentrated in low-intensity regions across different cumulative time values, while higher-intensity states appear dispersed throughout the domain. The resulting structure does not exhibit a proportional relationship between operational intensity and cumulative time, indicating non-linear behavior among the reconstructed state components.

### 4.3 Decision policy behavior: baseline vs. proposed model

Decision-making behavior is analyzed using the function *D*_*t*_ = π(*S*_*t*_), evaluating structural differences between a rule-based policy and a multivariable policy defined on the reconstructed state. The quantitative characterization of both policies is presented in Table 3.

**Table 3:** Comparative characterization of decision policy behavior in the reconstructed state space.

Policy/source	Decision rate	Activation variance	Mean inter-decision gap	State sensitivity	Similar-state instability
Baseline policy	0.1435	0.3687	4.8608	0.3465	0.2097
Proposed policy	0.1000	0.1652	7.6918	1.0000	0.2468
Baseline vs. proposed divergence	0.1992	–	–	–	–
BPI real decision (accepted)	0.0251	–	–	–	–

The decision rate differs between the two policies, reaching 0.1435 for the baseline and 0.1000 for the proposed policy. This difference is accompanied by an increase in the mean inter-decision gap from 4.8608 to 7.6918 events, indicating that the proposed policy activates decisions less frequently and over more separated regions of the reconstructed trajectories.

Activation variance decreases from 0.3687 in the baseline policy to 0.1652 in the proposed policy. This reduction indicates lower dispersion in activation behavior across trajectories, producing a more stable distribution of decision responses throughout the reconstructed operational space.

State sensitivity presents the largest difference between policies. The baseline reaches a value of 0.3465, whereas the proposed policy reaches 1.0000, corresponding to a complete variation in activation rates across state deciles. This behavior indicates that the proposed policy adapts more strongly to changes in the reconstructed operational conditions, while the threshold-based baseline remains concentrated within narrower activation regions.

The instability metric for similar states reaches 0.2097 in the baseline and 0.2468 in the proposed policy. The higher value observed in the multivariable policy indicates greater local variation in decision responses within neighboring regions of the reconstructed state space.

The divergence between policies, measured as the proportion of events in which both policies generate different decisions, reaches 0.1992. This value indicates that approximately one-fifth of the generated decisions do not coincide between the two policies, reflecting structural differences in the activation criteria defined over the reconstructed state space.

In the BPI set, the observed decision rate reaches 0.0251, remaining below the activation levels observed in both policies generated over EdNet-KT1. This value is reported as a reference for real operational behavior, without incorporating state-dependent metrics because a directly comparable representation of the reconstructed state space is not available for the BPI decision process.

**Figure 5:**
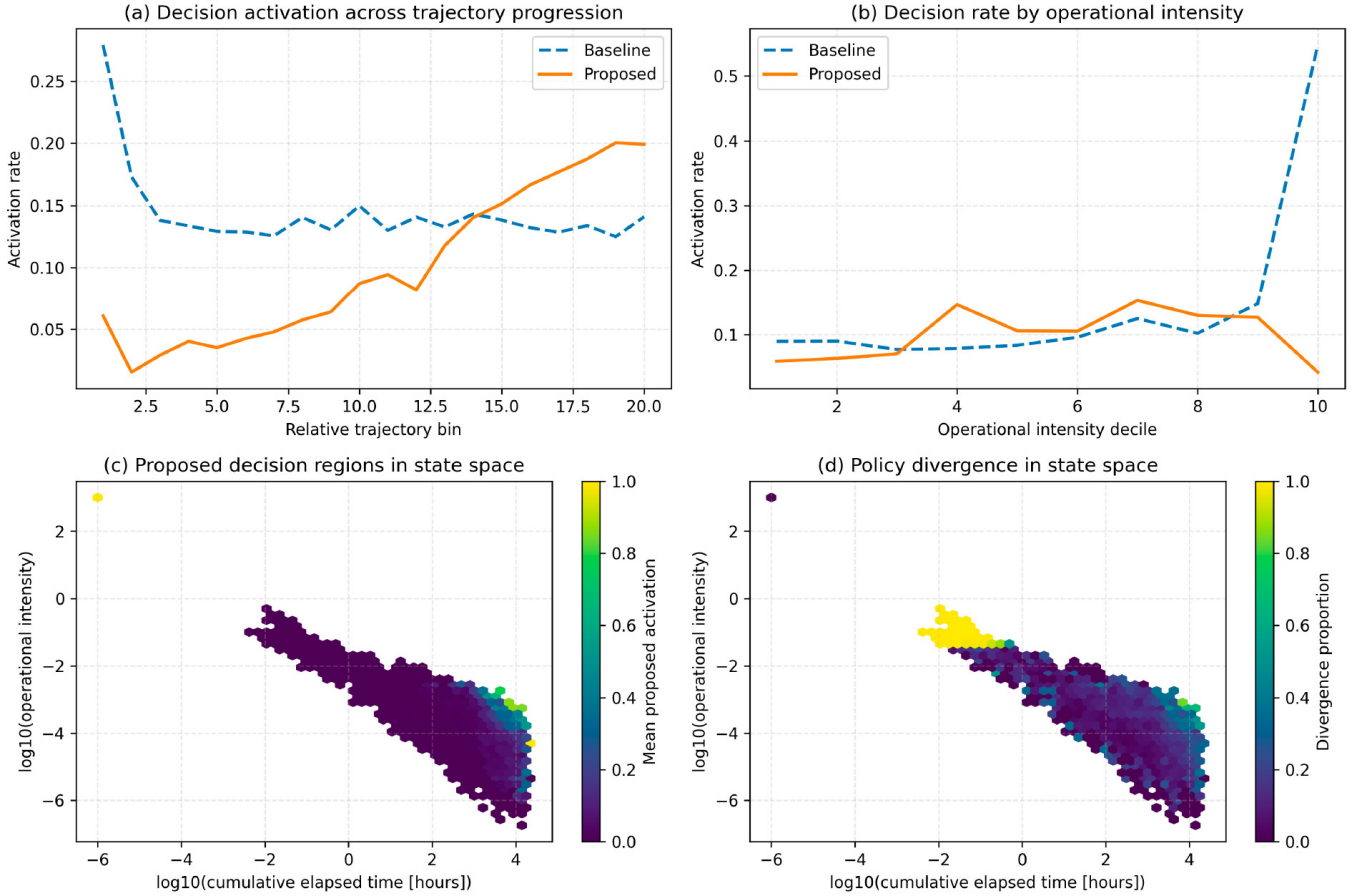
Behavior of decision policies in the reconstructed state space: **(a)** decision activation rate along the relative progression of the trajectory; **(b)** decision rate by operational intensity deciles; **(c)** activation regions of the proposed policy in the transformed space of accumulated time and operational intensity; **(d)** divergence map between policies.

Figure 5 presents the behavior of both policies across different operational domains. In Figure 5a, the activation rate is analyzed along the relative progression of the trajectories. The baseline policy exhibits elevated activation levels during the initial trajectory stages, followed by stabilization near 0.13. In contrast, the proposed policy progressively increases its activation rate throughout the trajectory, reaching comparable or higher values during later stages. This behavior indicates a temporal redistribution of decision activation across the reconstructed event sequence.

In Figure 5b, the decision rate is evaluated across operational intensity deciles. The baseline policy exhibits an abrupt increase in the highest decile, reaching activation values near 0.55 and concentrating decisions in the extreme-intensity region. The proposed policy instead shows a more gradual transition across the deciles, distributing activation behavior across intermediate-intensity ranges without a dominant increase at the upper boundary of the domain.

In Figure 5c, the activation behavior of the proposed policy appears concentrated within specific regions of the transformed state space, particularly where intermediate operational intensity and moderate accumulated time converge. The resulting distribution is not spatially uniform, indicating differentiated activation regions within the reconstructed operational domain.

In Figure 5d, the divergence between policies appears concentrated in localized regions of the transformed state space. These regions correspond to operational configurations in which the threshold-based and multivariable policies generate different responses under similar combinations of state variables. The resulting divergence structure is therefore associated with specific regions of the reconstructed domain rather than with uniformly distributed discrepancies.

### 4.4 State transition analysis and system response (Δ*S*_*t*_)

The sequential reconstruction of the state defines the transition dynamics, evaluating Δ*S*_*t*_ = *S*_*t*+1_−*S*_*t*_ as a function of the operational variables. The analysis is developed conditioned on the type of decision, differentiating between the absence of activation, activation by rules, and activation by multivariable policy, as presented in Table 4.

**Table 4:** State transition characterization under decision conditions.

Condition	Δ Intensity (mean)	Δ Intensity (median)	Δ Elapsed time (hours)	Δ Repeat rate
No decision	–0.000005	0.00000015	4.81	0.00029
Baseline decision	–24.62	0.00000011	6.57	–0.00046
Proposed decision	–35.06	0.00000005	2.29	–0.00952

The operational intensity variation (Δ) reaches a mean of −5.35 × 10^−6^ in the absence of decisions, with a median of 1.54 × 10^−7^, indicating transitions concentrated near zero magnitude. Under baseline activation, the mean decreases to −24.62, whereas the proposed policy reaches −35.06, while maintaining medians on the order of 10^−7^ in both conditions. The difference between the mean and the median indicates a highly asymmetric transition structure in which most state variations remain small. At the same time, a reduced subset concentrates larger reductions in operational intensity.

**Figure 6:**
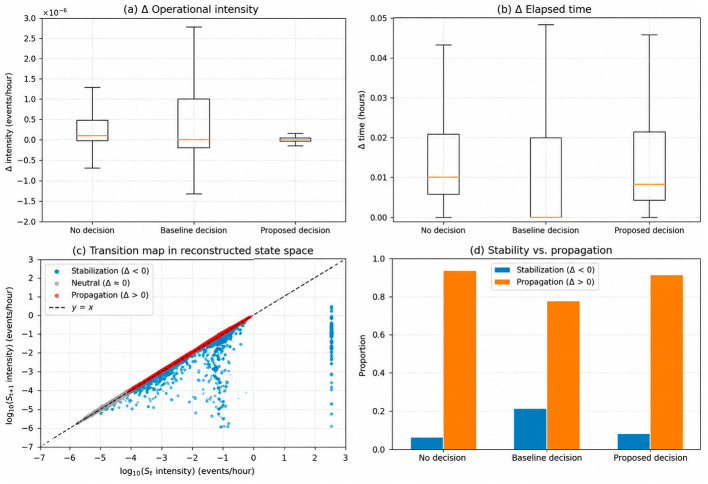
Transition dynamics of the reconstructed state under decision conditions: **(a)** distribution of Δ operational intensity by condition; **(b)** distribution of Δ elapsed time; **(c)** transition map in the state space log_10_(*S*_*t*_) vs. log_10_(*S*_*t*+1_); **(d)** proportion of stabilizing (Δ < 0) and propagating (Δ>0) transitions for each condition.

This behavior is reflected in Figure 6a, where the baseline policy exhibits broader negative tails across the intensity distribution. In contrast, the proposed policy concentrates transitions within narrower ranges, reducing the amplitude of extreme deviations while maintaining negative transitions throughout the reconstructed state space.

Elapsed time variation (Δ) reaches a mean of 4.81 h in the absence of decisions, increases to 6.57 h under the baseline policy, and decreases to 2.29 h under the proposed policy. This behavior indicates that transition timescales differ across the evaluated conditions: the baseline extends the temporal separation between consecutive states, whereas the proposed policy shortens the transition interval. In Figure 6b, the baseline distribution extends toward higher elapsed-time values, whereas the proposed policy concentrates most transitions within narrower temporal ranges.

The repeat question rate variation (Δ) remains close to zero in the absence of decisions (2.94 × 10^−4^) and under the baseline policy (−4.61 × 10^−4^), while the proposed policy reaches −9.52 × 10^−3^. Although the magnitude of these variations remains lower than during operational intensity transitions, the proposed policy yields a greater reduction in recurrence levels across the reconstructed trajectories.

The reconstructed transition space is represented in Figure 6c, where the relationship between log_10_(*S*_*t*_) and log_10_(*S*_*t*+1_) remains predominantly concentrated near the identity diagonal. This concentration indicates persistence between consecutive states across most trajectories. Negative transitions (Δ*S*_*t*_ < 0) appear distributed below the diagonal, mainly within intermediate state regions, while positive transitions remain closer to the diagonal, reflecting increases of lower magnitude.

The proportions of stabilizing and propagating transitions differ across the evaluated conditions. In the absence of decisions, transitions with Δ < 0 represent approximately 6% of the total, whereas transitions with Δ>0 exceed 90%. Under the baseline policy, the proportion of stabilizing transitions increases to approximately 21%, reducing the proportion of propagating transitions. The proposed policy also increases the proportion of stabilizing transitions relative to the no-decision condition while maintaining lower dispersion in transition magnitudes, as observed in Figure 6d.

### 4.5 Stability and adaptation under operational variability

System stability is assessed by the direction of transitions (Δ*S*_*t*_) as well as by the consistency of decisions across equivalent states, the policy's internal variability, and the capacity to adapt under different operating regimes. To this end, global and local performance metrics are integrated, and analyses are segmented by load and path length; the results are presented in Table 5.

**Table 5:** Decision consistency and intra-policy variability.

Policy	Decision rate	Activation variance	State sensitivity	Similar-state instability	Decision entropy
Baseline	0.142677	0.368943	0.379539	0.211659	0.591208
Proposed	0.100199	0.190314	1.000000	0.247356	0.469625

The proposed policy exhibits a decision rate of 0.1002, compared to 0.1427 in the baseline, and reduces activation variance from 0.3689 to 0.1903. This behavior indicates that the proposed policy activates decisions less frequently while maintaining lower dispersion in activation patterns across the reconstructed trajectories.

State sensitivity reaches its maximum value in the proposed policy (1.0), compared to 0.3795 in the baseline. This difference indicates that the activation probability in the multivariable policy varies substantially across different regions of the reconstructed state space, concentrating decisions within specific operational configurations rather than distributing them uniformly across trajectories.

Instability in similar states increases from 0.2117 to 0.2474 under the proposed policy, indicating greater local variability in activation behavior within neighboring state regions. At the same time, decision entropy decreases from 0.5912 to 0.4696, reflecting lower global uncertainty in the decision structure. The coexistence of lower entropy and higher local instability indicates that the proposed policy concentrates activation behavior in specific operational regions while preserving differentiated responses within similar state configurations.

The interaction between stability and adaptation is further analyzed under different operational regimes. Table 6 presents the behavior of both policies segmented by load level and trajectory type, allowing evaluation of the relationship between activation frequency, transition magnitude, and stabilization behavior across heterogeneous operational conditions.

**Table 6:** Adaptation under operational variability.

A	B	C	D	E	F	G	H
Low load	Baseline	0.088546	−1.08 × 10^−7^	0.000004	0.276018	0.107676	663
Low load	Proposed	0.057308	−4.22 × 10^−6^	0.000005	0.895161	0.061084	124
High load	Baseline	0.298534	–60.17	60.17	0.469565	0.318931	81,584
High load	Proposed	0.102797	–777.79	777.79	0.884646	0.220492	6,311
Short trajectories	Baseline	0.686523	–78.56	78.56	0.503613	0.294591	33,490
Short trajectories	Proposed	0.053921	–999.54	999.54	1.000000	0.237355	2,632
Long trajectories	Baseline	0.111933	–32.76	32.76	0.356025	0.101905	37,517
Long trajectories	Proposed	0.105053	–258.77	258.77	0.698673	0.065272	4,749

A, Condition; B, Policy; C, Decision rate; D, Δ intensity mean; E, Δ|intensity| mean; F, Stability rate; G, Activation variance; H, Activated events.

Under low-load conditions, the magnitude of Δ intensity remains on the order of 10^−6^, indicating a transition regime with minimal state variation. In this scenario, both policies exhibit limited influence on the reconstructed dynamics, although the proposed policy reaches a substantially higher stabilization rate (0.8952 vs. 0.2760).

Under high-load conditions, the difference between policies becomes more pronounced. The baseline shows an average intensity variation of −60.17, whereas the proposed policy shows −777.79, along with a higher stabilization rate (0.8846 vs. 0.4696). The larger negative transition magnitude should not be interpreted in isolation as evidence of improved stability. Instead, its operational significance emerges when considered jointly with the higher stabilization rate and lower activation variance observed under the proposed policy. These results indicate that the policy concentrates interventions within high-intensity operational regions and more frequently generates transitions directed toward reductions in operational intensity.

Short trajectories exhibit the largest contrast between policies. The baseline reaches a decision rate of 0.6865, whereas the proposed policy reduces activation to 0.0539. Despite this reduction, the proposed policy yields an average intensity variation of −999.54 and a stabilization rate of 1.0. These results indicate that a smaller number of activations is associated with transitions of greater magnitude and with a higher proportion of intensity-reducing transitions. Consequently, the observed stabilization behavior is associated with the direction and consistency of the transitions rather than with the magnitude of the transitions alone.

In long trajectories, these policies maintain similar decision rates (0.1119 for the baseline and 0.1051 for the proposed policy). In contrast, the proposed policy achieves substantially larger transition magnitudes (−258.77 vs. −32.76) and a higher stabilization rate (0.6987 vs. 0.3560). These results indicate that the difference between policies is associated not only with activation frequency but also with the direction and consistency of the resulting state transitions. The larger negative transition magnitudes observed under the proposed policy should not be interpreted in isolation as evidence of improved stability. Instead, their operational significance emerges when considered alongside the higher stabilization rate, indicating that a greater proportion of transitions are directed toward reducing operational intensity across the reconstructed trajectories.

## 5 Discussion

The results reveal structural differences between traditional operational approaches and the proposed framework, consistent with the fragmentation identified in the literature. Business intelligence systems prioritize process formalization and organizational traceability but generally operate under retrospective analytical schemes with limited adaptive capacity (Wu et al., 2024; Sequeira et al., 2025). Similarly, RPA architectures automate repetitive tasks through predefined rules without explicitly incorporating state-dependent decision behavior (Boza-Chua et al., 2022; Guarda et al., 2025). In contrast, the proposed framework integrates events, reconstructed states, and decision policies within a coupled temporal structure, enabling operational behavior to evolve dynamically as trajectories progress.

This difference becomes evident in the observed relationship between activation variance, state sensitivity, and transition behavior. While event-driven architectures improve responsiveness through continuous event processing (Eshankulov et al., 2026; Sowan et al., 2025), and multi-agent systems support distributed adaptation (Basingab, 2025; Peng et al., 2024), these approaches generally separate reactive processing, operational representation, and decision generation into independent layers. The proposed model instead evaluates these components jointly within a single operational structure, enabling direct analysis of how decisions affect the evolution of reconstructed trajectories.

From a methodological perspective, operational reconstruction from event logs transforms discrete observations into structured trajectories with observable state evolution. This formulation extends process-oriented reconstruction approaches (Bertrand et al., 2023) by integrating state-dependent decision policies directly into the reconstructed operational space. Because all variables are derived from observable event histories, the framework preserves traceability between raw interactions, reconstructed states, and generated decisions. Unlike process mining methodologies that primarily focus on process discovery, conformance analysis, or performance diagnostics, the proposed formulation explicitly incorporates decision-generation mechanisms into the reconstructed operational trajectories. Similarly, state transitions are evaluated as observable consequences of operational decisions rather than as reward-driven optimization processes, enabling direct analysis of how decision policies influence operational evolution over time.

The comparison between the baseline and the proposed policy demonstrates that the differences extend beyond activation frequency and affect the internal organization of operational behavior. Quantitatively, the proposed policy reduces the decision rate from 0.1427 to 0.1002 and decreases activation variance from 0.3689 to 0.1903, indicating lower global dispersion in activation behavior. At the same time, state sensitivity increases from 0.3795 to 1.0, indicating that the proposed formulation concentrates decisions within specific operational regions rather than uniformly distributing activations across trajectories.

This behavior persists under heterogeneous operational conditions. Under high-load regimes, the proposed policy produces substantially larger transition magnitudes than the baseline while maintaining lower activation variance and higher stabilization rates. Similarly, in short trajectories, the proposed model generates fewer activations but stronger stabilizing transitions. These results indicate a shift from frequent threshold-based interventions toward more selective state-dependent decision behavior with greater influence on system evolution.

The main contribution of the framework lies in formalizing operational intelligence as a measurable, dynamic process in which events, states, and decisions are jointly evaluated using observable operational metrics. Unlike approaches focused primarily on predictive accuracy or isolated automation efficiency, the proposed model enables analysis of emergent properties such as variability, stability, adaptation, and transition behavior within evolving trajectories (Cai et al., 2023). The empirical results show that the framework does not generate uniform changes across all operational conditions, but instead reorganizes decision behavior according to the structure of the reconstructed state space.

From an institutional perspective, this formulation enables the analysis of operational behavior beyond static workflow monitoring or descriptive analytics. Because the framework reconstructs trajectories directly from event logs while preserving temporal dependencies, it can support the evaluation of intervention patterns, operational bottlenecks, and adaptive decision behavior in environments characterized by heterogeneous activity flows.

For real-world operational deployment, the framework should be interpreted as a decision-support layer rather than as a fully autonomous decision-making mechanism. In institutional environments, generated activations would require human-in-the-loop validation by academic, administrative, or operational supervisors before being translated into formal actions. This validation layer is necessary to ensure that state-dependent recommendations remain consistent with institutional governance rules, accountability procedures, data-quality constraints, and domain-specific decision protocols. From an implementation perspective, the framework can be integrated with existing institutional platforms, such as learning management systems, workflow management systems, enterprise resource planning platforms, or operational dashboards, through event-log extraction and batch or streaming state reconstruction. Operational online adaptation could be supported through incremental recalibration of thresholds and state representations as new event trajectories are generated, while distributed deployment across departments, campuses, or organizational units would require synchronization mechanisms for heterogeneous event streams and consistent governance criteria across operational nodes. Such integration would enable the visualization and monitoring of reconstructed operational states and decision activations through institutional operational dashboards for supervisory analysis and intervention tracking.

However, the proposed framework also presents limitations. State reconstruction depends on temporal aggregation operators and sliding-window representations, which introduce scale dependence and may reduce sensitivity to fine-grained temporal variations. The policies are derived from empirical state distributions, preserving internal consistency but potentially limiting generalizability across operational domains with substantially different behavioral structures. In addition, the evaluation is conducted using EdNet-KT1 and BPI 2017. Although EdNet-KT1 originates from an educational environment, its use in this study is motivated by its operational characteristics rather than its pedagogical content, as it provides large-scale event trajectories with entity-level traceability, heterogeneous temporal behavior, and long-term sequential interactions that are required for state reconstruction and transition analysis. BPI 2017 complements the evaluation by providing an organizational process environment with real institutional workflows. Nevertheless, the operational characteristics of these datasets may not fully reflect those of other institutional environments, and additional validation in administrative and organizational settings remains necessary. Finally, the framework does not incorporate validation under real operational deployment with human intervention or organizational governance constraints, and the transition-based stability formulation represents only one dimension of system behavior.

In addition, the scalability of the framework under large-scale distributed operational environments was not evaluated in the present study. Although the proposed formulation preserves interpretability through explicit state reconstruction and observable operational variables, increasing the dimensionality and complexity of the reconstructed state space may reduce the transparency of multivariable decision behavior in more complex institutional deployments.

## 6 Conclusion

This study presents a formal operational intelligence framework in which events, reconstructed states, and decision policies are integrated within a coupled dynamic structure. The proposed formulation demonstrates that state-dependent multivariable policies modify not only the frequency of decision activation but also the internal organization of operational behavior throughout evolving trajectories.

The experimental results show that the proposed policy reduces the decision rate from 0.1427 to 0.1002 and decreases activation variance from 0.3689 to 0.1903, indicating lower global dispersion in activation behavior. At the same time, state sensitivity increases from 0.3795 to 1.0, demonstrating that the decision process becomes strongly dependent on specific operational configurations reconstructed from the event trajectories.

The transition analysis further reveals that the effects of the proposed policy vary according to the operational regime. Under high-load conditions, the model generates transition magnitudes as low as −777.79, compared to −60.17 in the baseline formulation. Similarly, in short trajectories, the proposed policy produces values close to −999.54 while maintaining a stabilization rate of 1.0. These results indicate that the proposed formulation shifts the intervention regime from frequent low-impact activations toward more selective decisions associated with larger operational transitions.

The proposed framework, therefore, does not optimize the system solely on conventional efficiency metrics. Instead, it reorganizes operational behavior by concentrating decision-making within critical regions of the reconstructed state space, enabling analysis of stability, variability, adaptation, and transition dynamics using observable operational metrics derived directly from event histories. By coupling event reconstruction, state evolution, and decision-generation mechanisms within a unified operational representation, the framework provides a formal basis for analyzing how operational decisions influence the evolution of institutional processes over time.

The study also demonstrates that institutional systems can be modeled as evaluable dynamic processes without incorporating exogenous variables or external supervisory information. Because the framework preserves traceability among events, reconstructed states, and generated decisions, it provides a reproducible basis for analyzing operational evolution in environments with heterogeneous temporal behavior.

Future research directions include validation in real operational environments with human intervention, the incorporation of adaptive learning mechanisms within the decision policy, and integration with distributed and multi-agent architectures to evaluate operational intelligence under more complex dynamic conditions.

## Data Availability

The original contributions presented in this study are included in the article and supplementary material. The datasets analyzed are publicly available and are cited in the manuscript. Additional materials related to data reconstruction, operational modeling, and experimental implementation are available from the corresponding author upon reasonable request.
